# Genome-Wide Identification and Characterization of the ABA Receptor *PYL* Gene Family in Broomcorn Millet (*Panicum miliaceum* L.)

**DOI:** 10.3390/biology15211944

**Published:** 2026-10-27

**Authors:** Yue Qiao, Ye Du, Zhiheng Wang, Huayong Shi, Junjie Wang

**Affiliations:** 1 Center for Agricultural Genetic Resources Research, Shanxi Agricultural University, Taiyuan 030031, China; 2 School of Economics and Management, Shanxi Agricultural University, Taiyuan 030006, China; 3 Taihang Mountain State-Owned Forest Management Bureau of Shanxi Province, Shanxi Forestry and Grassland Bureau, Jinzhong 030601, China; 4 College of Agriculture, Shanxi Agricultural University, Taigu, Jinzhong 030801, China

**Keywords:** broomcorn millet, PYL receptor, abiotic stress, qRT-PCR

## Abstract

Pyrabactin resistance-like (PYL) proteins are core abscisic acid (ABA) receptors, but their organization and stress-responsive functions remain poorly understood in broomcorn millet (*Panicum miliaceum* L.). Here, we identified *PmPYL* genes genome-wide and characterized their phylogeny, gene structure, conserved motifs, chromosomal distribution, synteny, selection pressure, promoter cis-elements, predicted regulatory networks, tissue expression, and responses to ABA, salinity, and osmotic stress. Fifteen *PmPYL* genes were identified, with eight located in the A subgenome and seven in the B subgenome. Phylogenetic analysis placed 11 PmPYL proteins in Group II and four in Group III, with none in Group I. Sequence and structural analyses revealed broad conservation of receptor features associated with ABA perception and PP2C interaction. Collinearity analysis identified seven synteny-supported A/B homoeologous pairs, while the examined duplicate pairs showed low Ka/Ks ratios. Promoter analyses revealed abundant hormone- and stress-responsive elements, while predicted protein interactions supported a conserved PYL–PP2C–SnRK2 signaling module. Pairwise promoter and tissue-expression comparisons indicated regulatory variation among retained homoeologs. qRT-PCR analysis of seedling leaves further showed distinct treatment- and time-dependent expression patterns, with *PmPYL10*, *PmPYL8*, and *PmPYL12* showing pronounced changes under the examined conditions. These findings reveal a conserved ABA receptor framework with substantial regulatory diversification and identify candidate *PmPYL* genes for functional analysis of stress adaptation in broomcorn millet.

## Simple Summary

Broomcorn millet is a drought-tolerant cereal that grows well in dry and marginal environments, but the molecular mechanisms supporting its stress adaptation are not fully understood. Abscisic acid is a plant hormone that helps plants respond to water shortage, salinity, and other stresses. In this study, we identified 15 genes encoding abscisic acid receptors in broomcorn millet and examined their evolutionary relationships, gene structures, regulatory features, tissue expression, and responses to hormone, salt, and osmotic treatments. The receptor proteins showed broad sequence conservation, whereas their corresponding genes differed in predicted promoter features and tissue-expression patterns. In seedling leaves, several genes showed pronounced changes in transcript abundance relative to the pretreatment baseline following ABA, salt, and osmotic treatments. These expression profiles identify candidates for further investigation of ABA signaling and stress responses in broomcorn millet.

## 1. Introduction

Broomcorn millet is one of the oldest domesticated cereal crops and has been cultivated for thousands of years in arid and semi-arid regions of Eurasia [1,2,3]. As a C_4_, short-season, and drought-tolerant grain, broomcorn millet is well adapted to marginal lands with low rainfall, poor soil fertility, and large temperature fluctuations [4,5]. In recent years, broomcorn millet has attracted renewed interest because of its excellent stress tolerance, high water-use efficiency, and nutritional value, including abundant dietary fiber, minerals, and bioactive compounds [6,7,8,9]. Despite its agronomic and nutritional importance, the molecular mechanisms underlying stress adaptation and hormone signaling in broomcorn millet remain far less understood than in model crops such as rice and *Arabidopsis*.

Abscisic acid (ABA) is a central phytohormone that coordinates plant growth, development, and adaptation to adverse environments. It regulates seed dormancy and germination, stomatal closure, root development, osmotic adjustment, and responses to drought and salinity [10,11,12]. The core ABA signaling pathway is initiated by PYRABACTIN RESISTANCE/PYRABACTIN RESISTANCE-LIKE/REGULATORY COMPONENT OF ABA RECEPTOR (PYR/PYL/RCAR) proteins. Upon ABA binding, these soluble receptors interact with and inhibit clade A protein phosphatase 2Cs (PP2Cs), thereby releasing SNF1-related protein kinase 2s (SnRK2s) from PP2C-mediated repression [11,13,14]. Activated SnRK2s subsequently phosphorylate ABA-responsive transcription factors, ion channels, and other downstream targets to reprogram gene expression and physiological responses [13,15]. The PYR/PYL/RCAR–PP2C–SnRK2 module is highly conserved in land plants and represents the central signaling framework through which changes in cellular ABA concentration are translated into developmental and stress responses [16,17].

Despite conservation of this core signaling pathway, individual PYL receptors can differ substantially in their expression patterns, ABA sensitivity, biochemical properties, and physiological functions. Genetic studies in *Arabidopsis* have demonstrated extensive functional redundancy among PYR/PYL/RCAR receptors while also revealing receptor-specific roles in stomatal regulation, root architecture, seed germination, and drought responses [18,19,20]. For example, PYL8 promotes lateral root growth through MYB77-dependent auxin-responsive transcription [20], whereas PYL9 contributes to drought resistance and ABA-associated leaf senescence [19]. Functional diversification is also evident in crops. Overexpression of *OsPYL5* and *OsPYL10* enhances drought or combined drought and cold tolerance in rice, although increased ABA sensitivity can also impose growth penalties [21,22]. Several rice PYL receptors additionally exhibit distinct ABA sensitivities and physiological functions [23], while *ZmPYL8*, *ZmPYL9*, and *ZmPYL12* contribute to drought resistance in maize [24]. More recently, genome-wide characterization and functional validation of *SiPYL3* in foxtail millet demonstrated that individual PYL receptors can exert contrasting effects under different stresses, further emphasizing that PYL-mediated stress regulation is both gene- and stress-dependent [25]. These findings indicate that PYL receptors function within finely balanced growth–stress regulatory networks rather than acting as uniformly positive regulators of stress tolerance.

Genome-wide analyses have identified PYL gene families in rice, maize, wheat, foxtail millet, cucumber, poplar, and other plant species [25,26,27,28,29,30]. These studies have revealed substantial variation in gene copy number, chromosomal organization, duplication history, and stress-responsive expression among species. Gene duplication is particularly important in the evolution of signaling gene families because duplicated receptors may retain conserved biochemical functions while diverging in their spatial or environmental regulation. This process may be especially relevant in broomcorn millet, an allotetraploid species whose genome retains extensive homoeologous relationships between its constituent subgenomes [3,6,31]. However, whether genome duplication has contributed to the retention and subsequent regulatory diversification of the PYL receptor repertoire in broomcorn millet remains unknown.

PYL receptor activity is regulated at multiple levels beyond gene copy number and protein sequence. Promoter architecture contributes to tissue- and stress-specific transcription, whereas interactions between ABA-bound PYL receptors and clade A PP2Cs determine the activation state of downstream SnRK2-dependent signaling [11,32]. ABA signaling is additionally controlled through feedback mechanisms that regulate the abundance and turnover of signaling components, thereby preventing prolonged or excessive pathway activation [24]. Despite this increasingly detailed understanding of PYL regulation in model and crop species, the composition, evolutionary history, and regulatory characteristics of the PYL family in broomcorn millet remain largely unexplored. In particular, it remains unclear how PYL genes have been retained following polyploidization, whether duplicated members have undergone regulatory differentiation, and which receptors respond most strongly to ABA, salinity, and osmotic stress. Addressing these questions is important for understanding how a highly conserved hormone-perception system has diversified in a crop adapted to dry and marginal environments.

In this study, we performed a genome-wide identification and systematic characterization of the PYL gene family in broomcorn millet. We investigated the chromosomal distribution, physicochemical properties, phylogenetic relationships, gene structures, conserved motifs, intra- and interspecific synteny, and evolutionary selection of *PmPYL* genes. Finally, time-course qRT-PCR was used to characterize the transcriptional responses of *PmPYL* genes to 50 and 100 μM ABA, 200 mM NaCl, and 20% PEG-6000-induced osmotic stress. By integrating evolutionary, regulatory, and expression evidence, this study provides a framework for understanding ABA receptor diversification in broomcorn millet and identifies stress-responsive *PmPYL* members for subsequent functional characterization.

## 2. Materials and Methods

### 2.1. Plant Materials and Stress Treatments

Broomcorn millet cultivar AJ 08, developed by the Center for Agricultural Genetic Resources Research, Shanxi Agricultural University (Shanxi, China), was used in this study. Seeds were surface-sterilized and germinated in Petri dishes containing distilled water for 48 h. Uniformly germinated seedlings were subsequently transferred to 21-cell cultivation trays (54 × 28 × 6.5 cm) filled with sterilized soil, with three seedlings grown in each cell (63 seedlings per tray), and maintained in a controlled-environment growth chamber under a 16 h light/8 h dark photoperiod at 25 °C until the three-leaf stage.

At the three-leaf stage, uniformly developed seedlings were subjected to osmotic, salt, and abscisic acid (ABA) treatments. Osmotic stress was imposed by irrigation with 20% (*w*/*v*) polyethylene glycol 6000 (PEG-6000), whereas salt stress was imposed by irrigation with 200 mM NaCl. For ABA treatment, seedlings were irrigated with either 50 μM or 100 μM ABA (Beijing Solarbio Science & Technology Co., Ltd., Beijing, China) solution. The ABA stock solution was prepared using 95% ethanol and stored at −20 °C protected from light. The final ethanol concentration in both ABA treatment solutions was 0.1% (*v*/*v*).

For the time-course experiment, separate cultivation trays were assigned to each treatment condition, sampling time, and biological replicate. The cultivation tray constituted the independent experimental unit, and trays were not randomly allocated. Young leaf samples were collected immediately before treatment (0 h) and at 3, 6, 12, and 24 h after treatment. At each sampling time, one individual plant was collected destructively from each of three independent trays, providing three biological replicates per treatment and time point. Each tray was sampled only once. The pretreatment 0 h samples served as baseline calibrators for subsequent relative expression analysis; no parallel time-matched untreated/mock controls were included. All samples were immediately frozen in liquid nitrogen and stored at −80 °C until RNA extraction.

### 2.2. Identification of PYL Genes in Broomcorn Millet and Other Plant Species

The broomcorn millet reference genome and its corresponding annotation files were retrieved from the National Center for Biotechnology Information (NCBI; https://www.ncbi.nlm.nih.gov/, accessed on 1 August 2026; GenBank accession GCA_038442765.1; BioProject PRJNA1059665. The rice (*Oryza sativa* L.) reference genome (IRGSP-1.0/RGAP Release 7) and its corresponding annotation files were retrieved from the Rice Genome Annotation Project (RGAP; https://rice.uga.edu/download_osa1r7.shtml, accessed on 1 August 2026). The *Arabidopsis thaliana* reference genome (TAIR10) and its corresponding annotation files were retrieved from The Arabidopsis Information Resource (TAIR; https://www.arabidopsis.org/download/list?dir=Genes/TAIR10_genome_release, accessed on 1 August 2026). The foxtail millet (*Setaria italica* L.) reference genome (v2.2) and its corresponding annotation files were retrieved from Phytozome (https://phytozome-next.jgi.doe.gov/, accessed on 1 August 2026). Similarly, the sorghum (*Sorghum bicolor* (L.) Moench) reference genome (v3.1.1) and its corresponding annotation files were retrieved from Phytozome (https://phytozome-next.jgi.doe.gov/, accessed on 1 August 2026).

To identify members of the *PYL* gene family, two complementary approaches were employed. First, known *A. thaliana* PYL protein sequences were used as queries for BLASTP (v2.15.0+) searches against the broomcorn millet protein database. Candidate proteins with an E-value < 1 × 10^−30^ and sequence identity > 50% were retained (Table S1). Second, the Hidden Markov Model (HMM) profile of the PYR/PYL/RCAR domain (PF10604) was retrieved from the Pfam database (http://pfam.xfam.org/, accessed on 1 August 2026) and used to search the broomcorn millet proteome. Candidate proteins obtained from the two approaches were combined, and redundant sequences were removed (Table S2). The conserved domains of the resulting candidate proteins were subsequently verified, and proteins containing a complete PYR/PYL/RCAR domain were retained as *PmPYL* family members (Table S3).

### 2.3. Chromosomal Distribution and Physicochemical Characterization of PmPYL Genes

The chromosomal positions of *PmPYL* genes were extracted from the broomcorn millet genome annotation file and visualized in R using the ggplot2 package [33]. Theoretical molecular weights and isoelectric points (pI) of the encoded proteins were calculated using the Peptides package (v2.4.6) in R.

Putative subcellular localization of PmPYL proteins was predicted using WoLF PSORT (https://wolfpsort.hgc.jp/, accessed on 5 August 2026). Protein secondary structures were predicted using the SOPMA algorithm implemented on the PRABI platform (https://npsa-prabi.ibcp.fr/, accessed on 5 August 2026).

### 2.4. Multiple Sequence Alignment and Phylogenetic Analysis

For comparative phylogenetic analysis, full-length PYL protein sequences from *Panicum miliaceum* [31], *Arabidopsis thaliana* [26], *Oryza sativa* [26], *Setaria italica* [26], and *Sorghum bicolor* [26] were aligned using MAFFT (v7) with the -auto option. Maximum-likelihood (ML) phylogenetic analysis was performed using IQ-TREE 2 (v2.2.3) based on the resulting alignment of 59 protein sequences (344 amino acid positions) [34]. The best-fitting amino acid substitution model (JTT + I + G4) was selected using ModelFinder implemented in IQ-TREE 2 (v2.2.3) according to the Bayesian information criterion (BIC). Branch support was assessed using 1000 SH-like approximate likelihood ratio test (SH-aLRT) replicates and 1000 ultrafast bootstrap replicates, with additional bootstrap refinement using the -bnni option [35]. The resulting phylogenetic tree was visualized in circular format using the Interactive Tree of Life (iTOL; https://itol.embl.de/, accessed on 5 August 2026), and the major phylogenetic groups were distinguished according to their clustering patterns (Table S4).

To examine sequence conservation among the 15 PmPYL proteins, an independent multiple sequence alignment was performed in R using the msa package (v1.24.0) with the ClustalOmega algorithm. The alignment was visualized using the msaPrettyPrint() function with the TeXshade output format. The characteristic gate and latch motifs were identified and annotated according to their aligned amino acid residues.

### 2.5. Gene Structure and Conserved Motif Analysis

The exon–intron structures of *PmPYL* genes were determined based on the broomcorn millet genome annotation file. Conserved motifs in PmPYL proteins were identified using MEME Suite (v5.5.5), with the maximum number of motifs set to 10 and motif lengths ranging from 6 to 200 amino acids; all other parameters were kept at their default settings.

Gene structures and conserved motif distributions were integrated and visualized using the Gene Structure Display Server (GSDS 2.0; https://gsds.gao-lab.org/, accessed on 5 August 2026), allowing direct comparison of exon–intron organization and motif architecture among individual *PmPYL* members.

### 2.6. Collinearity, Gene Duplication, and Evolutionary Analysis

Intra-genomic collinearity analysis was performed using MCScanX (v1.0.0) based on the broomcorn millet genome annotation and protein sequence files [36]. MCScanX was run using its recommended default parameters to identify collinear blocks and duplicated gene pairs. Collinear blocks containing *PmPYL* loci were extracted to evaluate duplication patterns within the family.

A/B homoeologous pairs were distinguished from other collinear duplicates by direct PYL–PYL anchors in MCScanX blocks linking corresponding A- and B-subgenome chromosomes, supported by protein similarity and conserved flanking genes. Corresponding regions were inspected for PmPYL loci without an annotated counterpart (Table S5).

For comparative synteny analysis, orthologous relationships between broomcorn millet and representative plant species were identified based on their genome annotation and protein sequence datasets. Syntenic relationships involving *PYL* loci were extracted and visualized to assess conservation between broomcorn millet and other species.

To evaluate evolutionary selection, coding sequences of duplicated *PmPYL* gene pairs were aligned in-frame using MAFFT version 7 [37]. Nonsynonymous substitution rates (Ka), synonymous substitution rates (Ks), and Ka/Ks ratios were calculated using KaKs_Calculator 3.0 under the Yang–Nielsen model [38].

Divergence times of duplicated gene pairs were estimated using:

T = K_s_/(2r) × 10^−9^

where *T* represents divergence time in million years ago (MYA), *Ks* is the synonymous substitution rate, and *r* is the neutral substitution rate of 6.5 × 10^−9^ substitutions per site per year [39,40].

### 2.7. Cis-Acting Regulatory Element Analysis

To investigate potential transcriptional regulation of *PmPYL* genes, the 2000 bp genomic sequences upstream of the translation initiation codon of each gene were extracted and defined as putative promoter regions. Cis-acting regulatory elements were identified using the PlantCARE database (http://bioinformatics.psb.ugent.be/webtools/plantcare/html/, accessed on 15 August 2026). Identified elements were classified according to their reported functional categories, and their distribution and frequency were summarized and visualized in R using ggplot2 (Table S6).

For the seven synteny-supported A/B pairs, promoter composition was compared using presence/absence-based Jaccard dissimilarity of named non-core PlantCARE motif types after excluding TATA/CAAT boxes and uninformative entries.

### 2.8. Tissue-Specific Expression Analysis of PmPYL Genes

To characterize the tissue-specific expression patterns of *PmPYL* genes, we analyzed the publicly available developmental RNA-seq dataset of broomcorn millet reported by Zou et al. [6] (NCBI BioProject PRJNA431485). The dataset covers 1-week-old seedlings, 3-week-old shoots, 8-week-old leaf blades, leaf sheaths, stems, inflorescences and roots, and 12-week-old mature seeds. Expression values in the published dataset were reported as RPKM.

To associate genes in the expression matrix with AJ8 PmPYL loci, AJ8 PmPYL protein sequences were compared with proteins from the earlier assembly using BLASTP. The AJ8 IDs, corresponding expression-reference IDs, and sequence matches are provided in Table S7.

For homoeolog comparisons, the eight tissue/stage RPKM values were transformed as log_2_ (RPKM + 1); pairwise Pearson correlations and root mean square errors (RMSE) described expression-pattern similarity and magnitude differences. An exploratory exact comparison used all 24 one-to-one A/B mappings constrained within the three phylogenetic groups, with one-sided tests for lower promoter dissimilarity and higher expression correlation in the observed pairs. The two primary *p* values were Holm-adjusted.

### 2.9. Prediction of PmPYL Protein Interaction Networks

Protein association data for *A. thaliana* PYL receptors and related ABA signaling components were obtained from STRING version 12.0 (https://string-db.org/, accessed on 15 August 2026) [41]. Interactions with a combined confidence score ≥ 0.4 were retained as medium-confidence associations. Candidate *Arabidopsis* PYL matches for the PmPYL proteins represented in the network were identified by protein-sequence comparison; the corresponding protein IDs and alignment statistics are provided in Table S8.

### 2.10. Quantitative Real-Time PCR Analysis

Total RNA was extracted from leaf samples collected after ABA, NaCl, and PEG-induced osmotic treatments using the Polysaccharide and Polyphenol Plant Total RNA Extraction Kit (Jianstone, Beijing, China, TR225-D) according to the manufacturer’s instructions. RNA concentration and purity were assessed using an FC-1100 ultra-micro spectrophotometer (Suizhen, Hangzhou, China), and RNA integrity was examined by agarose gel electrophoresis.

First-strand cDNA was synthesized from 1 μg of total RNA using the UEIris RT Mix with DNase (All-in-One) Kit (UE, Suzhou, China, R2020) according to the manufacturer’s instructions. The 20 μL reverse transcription reaction contained 4 μL of 5× UEIris II RT MasterMix, 1 μL of dsDNase, up to 1 μg RNA, and nuclease-free water. The reaction was performed at 37 °C for 2 min, 55 °C for 15 min, and 85 °C for 5 min. The resulting cDNA was stored at −20 °C until further analysis.

Quantitative real-time PCR (qRT-PCR) was performed using an ABI StepOnePlus Real-Time PCR System (Applied Biosystems, Foster City, CA, USA) and StarLighter SYBR Green qPCR Mix (Universal) (Qihengxing, Beijing, China, FS-Q1002). Each 20 μL reaction contained 2 μL of 1:20-diluted cDNA, 10 μL of 2× SYBR Green Mix, 0.4 μL each of forward and reverse primers, and 7.2 μL of nuclease-free water. Amplification was performed at 98 °C for 30 s, followed by 40 cycles of 98 °C for 15 s and 60 °C for 30 s. A melting-curve analysis was subsequently performed to verify amplification specificity. A six-point, tenfold serial dilution of the cDNA stock (undiluted, 1:10, 1:100, 1:1000, 1:10,000, and 1:100,000) was used to generate a standard curve for *PmActin*. Ct values were plotted against the log10 of relative cDNA concentration. Amplification efficiency was calculated as E (%) = (10^(−1/slope)^ − 1) × 100. The measured slope, R^2^, and efficiency are provided in Table S9. Standard curves were not generated for the *PmPYL* target assays, and the expression stability of *PmActin* across treatments was not independently evaluated.

Three independent biological replicates were analyzed for each post-treatment condition and sampling time. Each replicate consisted of leaf tissue from one plant sampled destructively from a separate cultivation tray; the tray was the independent experimental unit. Three pretreatment samples provided a shared 0 h baseline. *PmActin* was used as the internal reference gene. Where multiple technical Ct readings were available, they were averaged within each biological replicate before ΔCt was calculated as the target-gene Ct minus the corresponding *PmActin* Ct. For each gene, the mean ΔCt of the three shared 0 h samples was used as the calibrator. Relative expression was calculated as 2^−ΔΔCt^ and is presented as mean ± SD of three biological replicates. Gene-specific primers were designed using Primer Premier 5.0 with attention to sequence differences among closely related *PmPYL* homologs. The 14 genes analyzed were *PmPYL1*–*PmPYL13* and *PmPYL15*; *PmPYL14* was excluded because repeat attempts did not yield a validated, reproducible qRT-PCR dataset. AJ8 locus IDs and primer sequences for the analyzed genes and *PmActin* are provided in Table S9.

Statistical inference was performed on biological-replicate ΔCt values separately for each gene. Post-treatment values were analyzed using a fixed-effects two-way ANOVA with treatment (50 μM ABA, 100 μM ABA, 200 mM NaCl, or 20% PEG-6000), sampling time (3, 6, 12, or 24 h), and their interaction. These were omnibus tests; the three shared pretreatment samples were excluded from the two-way model. In separate planned comparisons, each of the 16 treatment–time groups was compared with the shared 0 h group using a two-sided Dunnett procedure. Multiplicity adjustment covered the 16 comparisons within each gene, and adjusted *p* < 0.05 was considered significant. Model residuals were assessed for normality, and group variances were examined for homogeneity. The available raw Ct readings and sample identifiers are provided in Table S10.

## 3. Results

### 3.1. Genome-Wide Identification and Characterization of the PmPYL Gene Family

**Figure 1:**
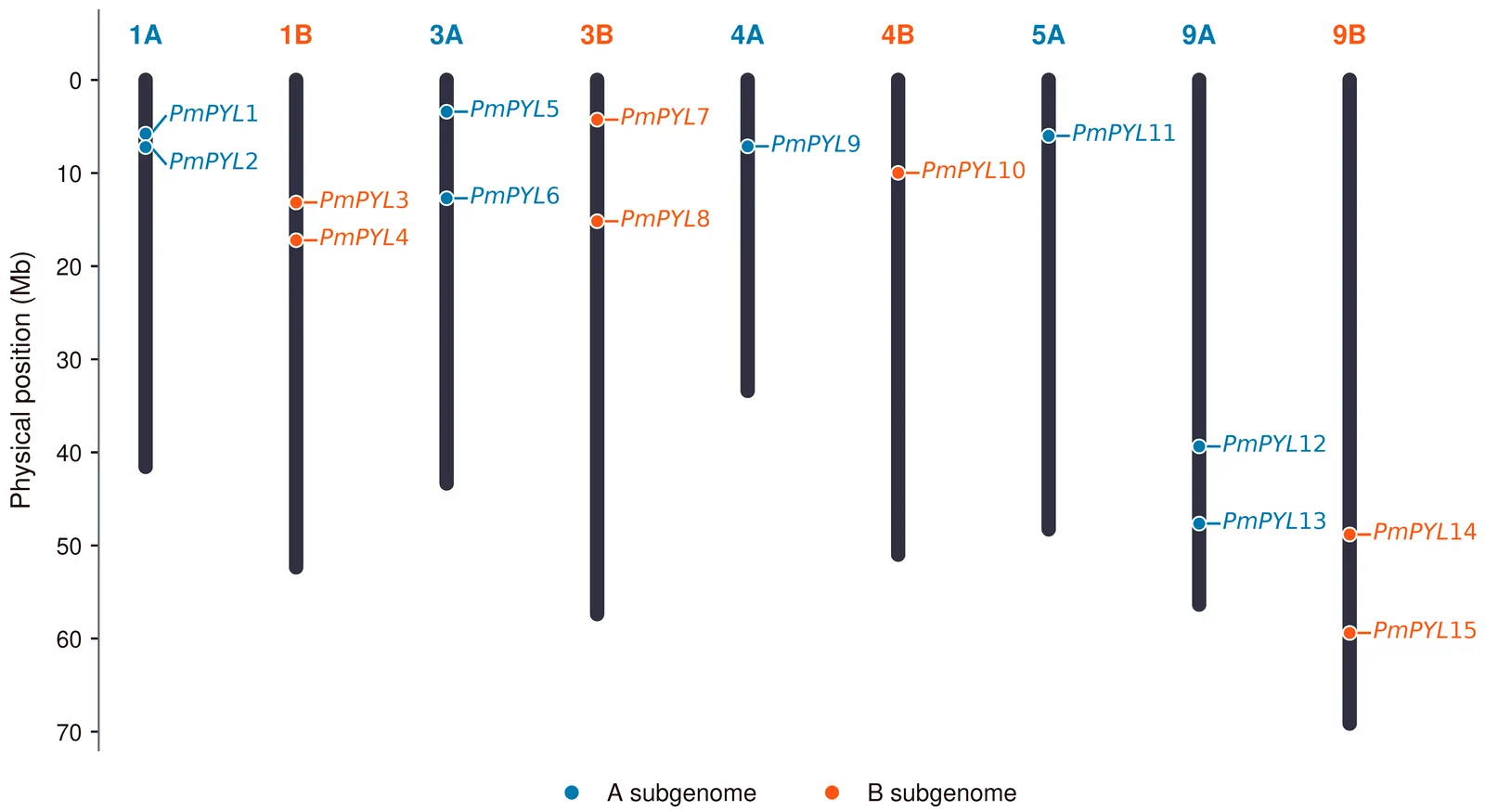
Chromosomal distribution of *PmPYL* genes in the AJ8 broomcorn millet genome. Only the nine chromosomes carrying the 15 identified *PmPYL* genes are shown. Grey bars are proportional to chromosome physical length, and circles indicate gene positions based on the genomic start coordinates in Table 1. Blue and orange denote genes on the A and B subgenomes, respectively. The vertical axis shows physical position in megabases (Mb).

In the AJ8 reference genome, a total of 15 PmPYL genes were mapped to chromosomes 1, 3, 4, 5 and 9 of the A and B subgenomes (Figure 1). Chromosomes 1A, 3A, 3B, 9A and 9B each harbor two PmPYL genes, whereas chromosomes 1B, 4A, 4B and 5A contain one or two members, and no PmPYL loci were detected on chromosomes 2, 6, 7 or 8. At the subgenome level, eight PmPYL genes are located on the A subgenome and seven on the B subgenome, indicating an overall balanced distribution. Most PmPYL genes occur as pairs on homoeologous A/B chromosome arms (1A–1B, 3A–3B, 9A–9B), suggesting relatively coordinated retention and divergence of the PmPYL family following genome duplication.

A total of 15 *PmPYL* genes were identified in the broomcorn millet genome (Table 1). The encoded proteins ranged from 196 to 223 amino acids in length, with predicted molecular weights of 21.26–24.02 kDa. Their theoretical isoelectric points ranged from 5.08 to 8.40. Most PmPYL proteins were neutral to slightly acidic, whereas PmPYL6 and PmPYL8 were relatively basic, with predicted pI values of 8.16 and 8.40, respectively.

### 3.2. Sequence Conservation and Phylogenetic Relationships of PmPYL Proteins

**Figure 2:**
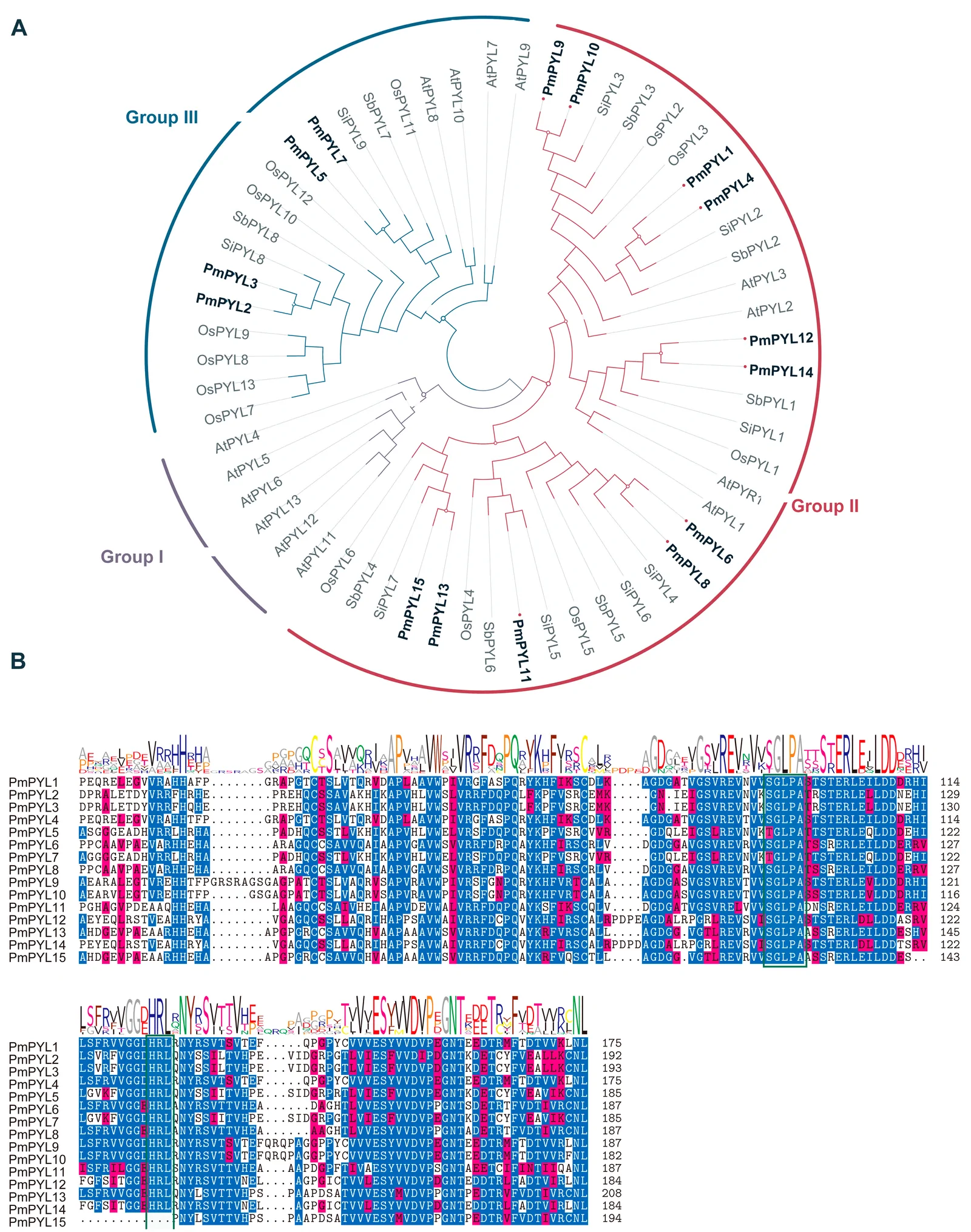
Phylogenetic relationships and sequence conservation of PYL proteins in broomcorn millet and other plant species. (**A**). Maximum-likelihood phylogenetic tree constructed using 59 full-length PYL protein sequences from broomcorn millet (*Panicum miliaceum*), *Arabidopsis thaliana*, rice (*Oryza sativa*), foxtail millet (*Setaria italica*), and sorghum (*Sorghum bicolor*). The proteins were classified into three major phylogenetic groups (Groups I–III), distinguished by different branch colors and outer arcs. PmPYL proteins are highlighted in bold. (**B**). Multiple sequence alignment of the 15 PmPYL proteins. Amino acid conservation is indicated by residue-specific shading and sequence logos. The characteristic gate (SGLPA/TGLPA) and latch (HRL) motifs are highlighted by green boxes.

Phylogenetic relationships were further examined using 59 PYL proteins from broomcorn millet, *Arabidopsis thaliana*, *Oryza sativa*, *Setaria italica*, and *Sorghum bicolor*. The maximum-likelihood tree was presented in three color-coded phylogenetic groupings (Figure 2A). Group I included six *Arabidopsis* proteins (AtPYL4, AtPYL5, AtPYL6, AtPYL11, AtPYL12, and AtPYL13) and no PmPYL members. Group II encompassed 34 proteins in the displayed tree, including 11 PmPYLs (PmPYL1, PmPYL4, PmPYL6, PmPYL8, PmPYL9, PmPYL10, PmPYL11, PmPYL12, PmPYL13, PmPYL14, and PmPYL15). Group III contained 19 proteins, including the remaining four PmPYLs (PmPYL2, PmPYL3, PmPYL5, and PmPYL7).

To further illustrate the evolutionary relationships among broomcorn millet PYL proteins, the 15 PmPYL members were extracted from the five-species ML phylogeny and presented separately (Figure S1). All seven synteny-supported A/B homoeologous pairs formed sister relationships: PmPYL1/PmPYL4, PmPYL2/PmPYL3, PmPYL5/PmPYL7, PmPYL6/PmPYL8, PmPYL9/PmPYL10, PmPYL12/PmPYL14, and PmPYL13/PmPYL15. The corresponding sister branches received SH-aLRT support values ranging from 77.8% to 95.2% and ultrafast bootstrap values ranging from 84% to 100%. These phylogenetic relationships were consistent with the synteny-based identification of A/B homoeologous pairs.

Multiple sequence alignment revealed broad conservation of the characteristic gate and latch regions among the 15 PmPYL proteins (Figure 2B). Thirteen proteins contained the SGLPA gate motif, whereas PmPYL5 and PmPYL7 carried a TGLPA variant. The HRL latch motif was observed in 14 proteins; the current PmPYL15 annotation lacked the corresponding aligned HRL segment.

### 3.3. Predicted Secondary Structure and Subcellular Localization of PmPYL Proteins

The predicted secondary-structure composition and subcellular localization of the 15 PmPYL proteins are summarized in Table 2. The proportions of α-helices ranged from 27.27% to 34.29%, extended strands from 25.23% to 36.18%, and random coils from 31.16% to 43.20%. Random coils generally accounted for the largest proportion of the predicted secondary structures, although moderate variation was observed among individual PmPYL proteins.

Subcellular localization prediction revealed considerable variation among PmPYL family members. Seven proteins (PmPYL1, PmPYL2, PmPYL3, PmPYL5, PmPYL7, PmPYL11, and PmPYL12) were predicted to localize predominantly in the cytoplasm. PmPYL4 was predicted to localize to both the nucleus and cytoplasm, whereas PmPYL6, PmPYL8, and PmPYL14 were predicted to localize to chloroplasts. PmPYL9 and PmPYL10 showed predicted dual localization to chloroplasts and the cytoplasm, while PmPYL13 and PmPYL15 were predicted to localize to the extracellular space and/or vacuole. These assignments are based solely on computational analysis. In particular, the chloroplast and extracellular/vacuolar predictions should be regarded as tentative and require independent experimental validation, such as fluorescent-protein localization.

### 3.4. Gene Structure and Conserved Motif Composition of PmPYL Genes

**Figure 3:**
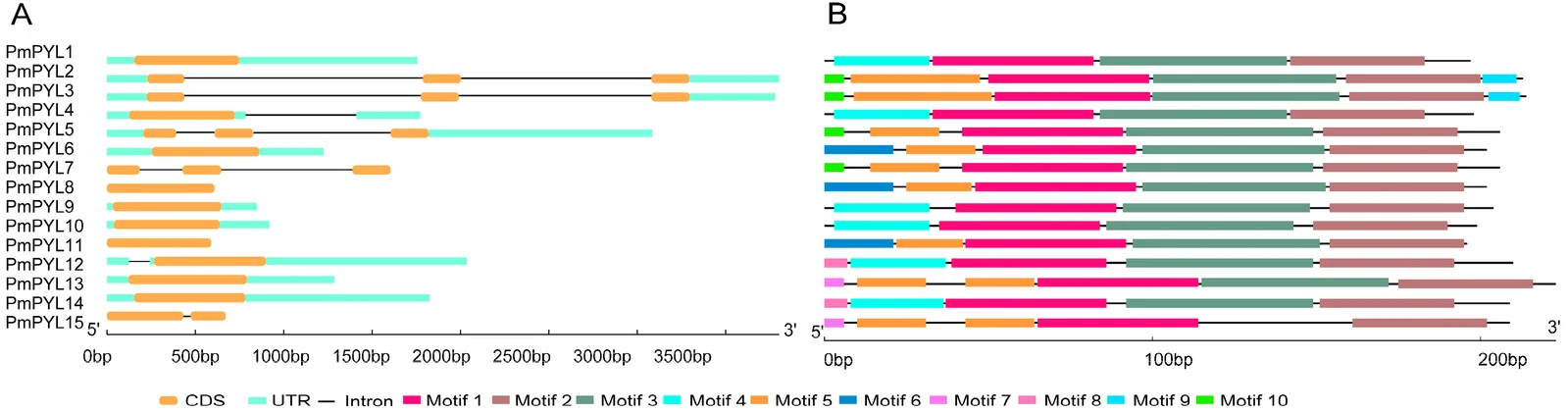
Gene structure and conserved motif composition of *PmPYL* genes. (**A**). Exon–intron organization of the 15 *PmPYL* genes based on genomic and coding sequences. Exons (CDS) are shown as orange boxes, introns as black lines, and untranslated regions (UTRs) as light green boxes. (**B**). Distribution of conserved motifs in PmPYL proteins identified using MEME. Ten conserved motifs (Motifs 1–10) are represented by colored boxes.

Analysis of exon–intron organization showed that most *PmPYL* genes contained two to three exons, although intron length varied among individual members (Figure 3A). Genes belonging to the same phylogenetic group generally exhibited similar exon–intron structures.

A total of ten conserved motifs were identified in the 15 PmPYL proteins using MEME (Figure 3B). Most members shared similar motif compositions and arrangements, particularly Motifs 1, 3, and 5, which were present in nearly all proteins. However, slight differences in motif composition were observed in several members, including *PmPYL11* and *PmPYL13*. Overall, the similarity in exon–intron organization and motif distribution among closely related *PmPYL* members was consistent with their phylogenetic relationships.

### 3.5. Intra- and Interspecific Synteny Analysis of PmPYL Genes

**Figure 4:**
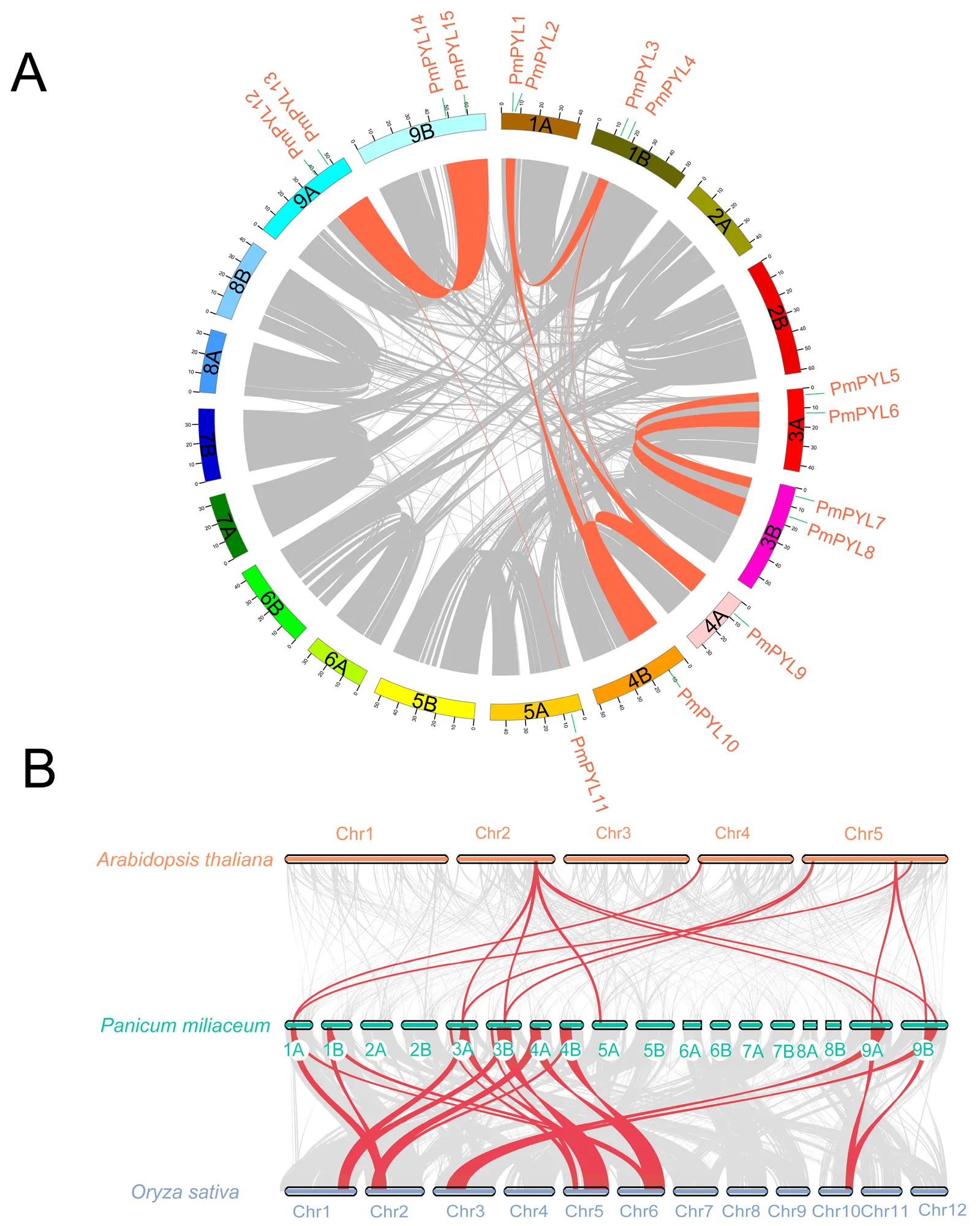
Intra- and interspecific synteny relationships of *PmPYL* genes. (**A**). Circos plot showing the chromosomal distribution and intra-genomic collinearity of *PmPYL* genes in broomcorn millet. The outer circle represents the 18 chromosomes (1A–9B). Red labels indicate *PmPYL* loci, red ribbons connect duplicated chromosomal regions containing *PmPYL* gene pairs, and grey lines represent genome-wide collinear blocks. (**B**). Comparative synteny of PYL genes among *Arabidopsis thaliana*, broomcorn millet (*Panicum miliaceum*), and rice (*Oryza sativa*). Chromosomes of the three species are arranged horizontally, and red curves connect syntenic PYL gene pairs across species.

Intra-genomic synteny analysis revealed multiple collinear relationships involving *PmPYL* loci across the broomcorn millet genome (Figure 4A). Several duplicated *PmPYL* gene pairs were located within conserved chromosomal blocks connecting the A and B subgenomes, particularly among chromosomes 1A/1B, 3A/3B, 4A/4B, and 9A/9B.

Seven synteny-supported A/B homoeologous pairs were resolved: *PmPYL1*–*PmPYL4*, *PmPYL2*–*PmPYL3*, *PmPYL5*–*PmPYL7*, *PmPYL6*–*PmPYL8*, *PmPYL9*–*PmPYL10*, *PmPYL12*–*PmPYL14* and *PmPYL13*–*PmPYL15* (Table S3). Thus, counterparts were detected for seven of eight A-subgenome members and all seven B-subgenome members. *PmPYL11* was unpaired; although flanking 5A/5B anchors identified the corresponding B-subgenome region, no annotated PYL counterpart was found there.

Comparative synteny analysis further revealed conserved PYL-associated genomic regions among broomcorn millet, rice, and *Arabidopsis* (Figure 4B). More syntenic relationships were detected between broomcorn millet and rice than between broomcorn millet and *Arabidopsis*. In broomcorn millet, conserved PYL-associated blocks were mainly located on chromosomes 1A/1B, 3A/3B, 4A/4B, and 9A/9B and corresponded to regions on rice chromosomes 1, 2, 3, 5, 6, and 10. Overall, these results demonstrate extensive intra-genomic duplication of *PmPYL* genes and stronger conservation of PYL-associated syntenic regions between broomcorn millet and rice.

### 3.6. Selection Pressure and Divergence Time of Duplicated PmPYL Gene Pairs

**Figure 5:**
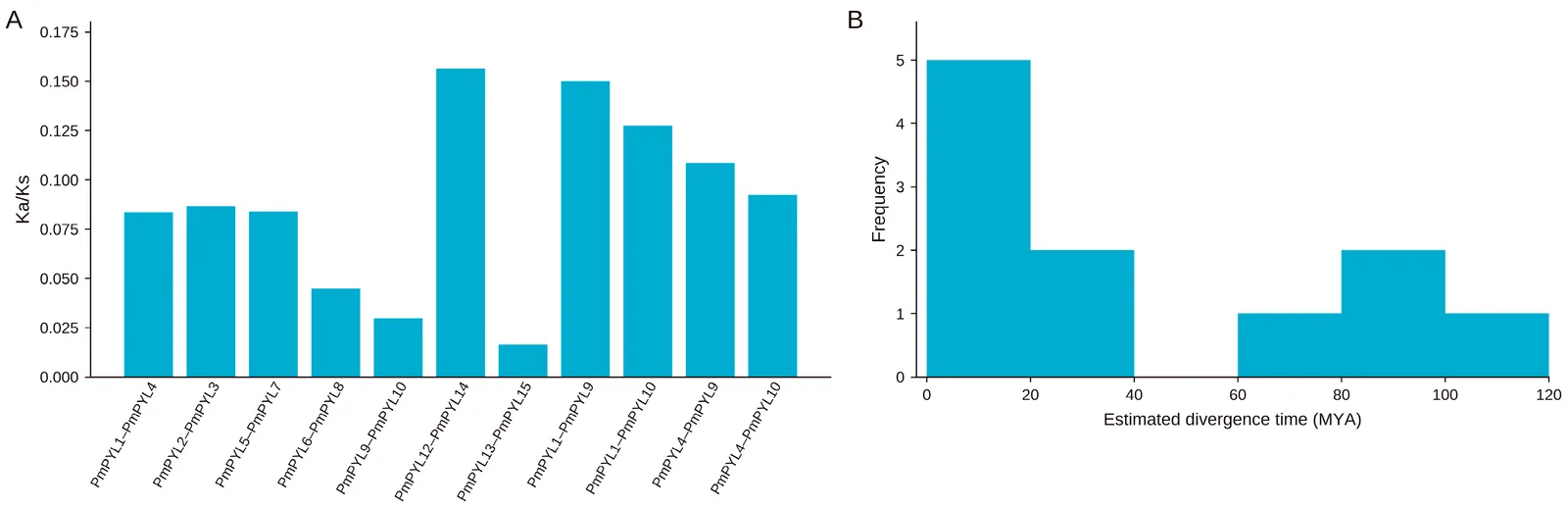
Molecular evolutionary analysis of collinear PmPYL gene pairs in broomcorn millet. (**A**) Ka/Ks ratios of the 11 collinear relationships included in the divergence-time analysis. (**B**) Frequency distribution of their approximate divergence times, calculated using a grass reference synonymous substitution rate of 6.5 × 10^−9^ substitutions per synonymous site per year.

Molecular evolutionary analysis was performed for the 11 PmPYL–PmPYL collinear relationships (Table 3; Figure 5A). The reported Ka/Ks ratios ranged from 0.0166 to 0.1563, with all values below 1.0, suggesting that purifying selection has contributed to the evolutionary conservation of these gene pairs.

Using the grass reference synonymous substitution rate of 6.5 × 10^−9^ substitutions per synonymous site per year, approximate divergence times were recalculated from the existing Ks values. The seven A/B homoeologous pairs yielded estimates ranging from approximately 5.1 to 23.7 MYA, whereas four additional non-homoeologous collinear relationships with retained dating estimates yielded values ranging from approximately 68.9 to 104.9 MYA (Figure 5B; Table 3).

### 3.7. Cis-Regulatory Elements in the Promoters of PmPYL Genes

**Figure 6:**
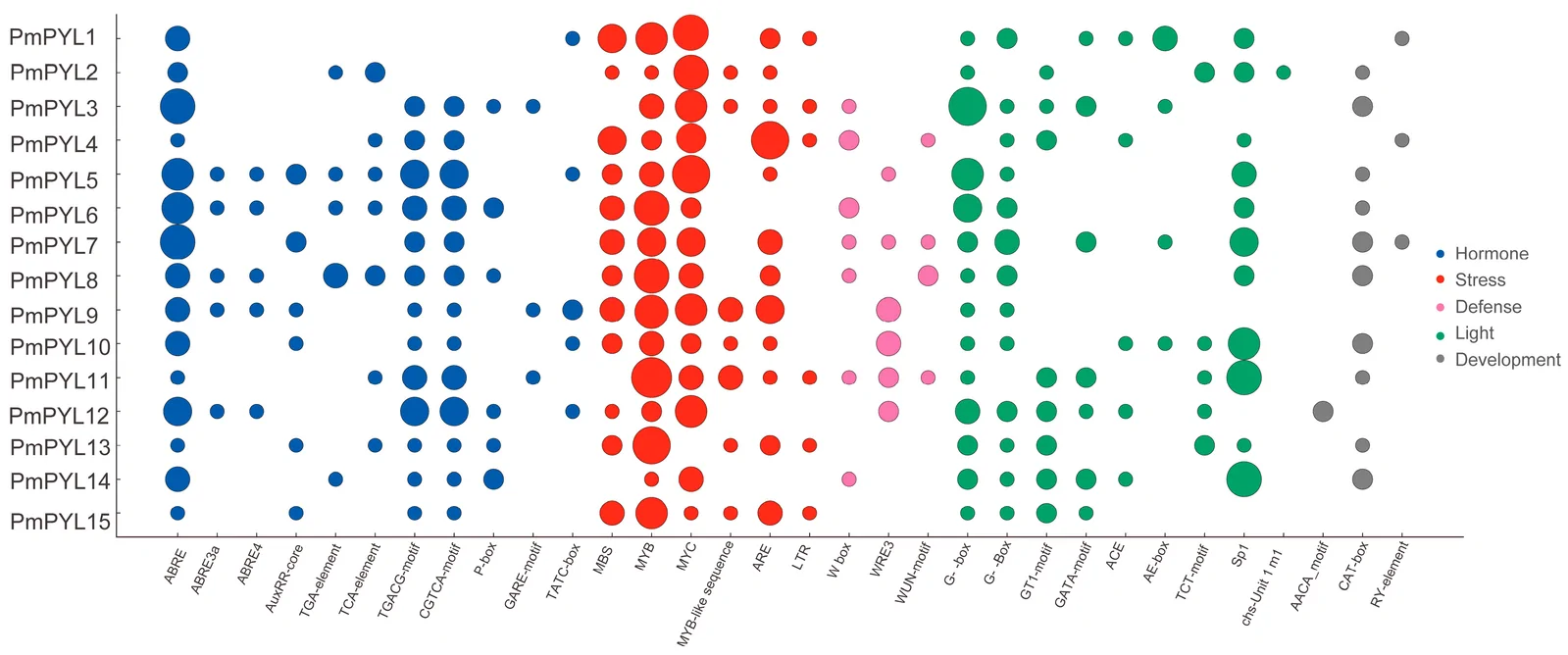
Distribution of cis-acting regulatory elements in the promoter regions of *PmPYL* genes. Bubble plot showing the distribution of cis-acting regulatory elements identified within the 2 kb upstream regions of the 15 *PmPYL* genes. Each row represents a *PmPYL* gene and each column represents a cis-element type. Bubble size indicates the number of occurrences of each element, and colors denote five functional categories: hormone-responsive, stress-responsive, defense-related, light-responsive, and development-related elements.

Analysis of the 2 kb upstream regions of the 15 *PmPYL* genes identified diverse cis-acting regulatory elements that were classified into five major functional categories: hormone-responsive, stress-responsive, defense-related, light-responsive, and development-related elements (Figure 6; Table S6). These annotations indicate potential regulatory sites but do not demonstrate transcription-factor binding or establish that the elements are active under the conditions examined.

Hormone-responsive elements were widely represented and included ABA-responsive elements (ABRE and related variants), auxin-responsive elements (AuxRR-core and TGA-element), gibberellin-responsive elements (P-box, GARE-motif, and TATC-box), and jasmonic acid- and salicylic acid-associated elements (TGACG-motif, CGTCA-motif, and TCA-element). Stress-associated elements included MBS, MYB, MYC, LTR, and ARE motifs. Defense-related elements, including W-box, WUN-motif, and WRE3, were detected in several promoters. Light-responsive elements, such as G-box, GT1-motif, GATA-motif, AE-box, ACE, and TCT-motif, were also broadly distributed, while development-associated elements included CAT-box, RY-element, and AACA_motif.

### 3.8. Predicted PmPYL-Associated Protein Interaction Network

**Figure 7:**
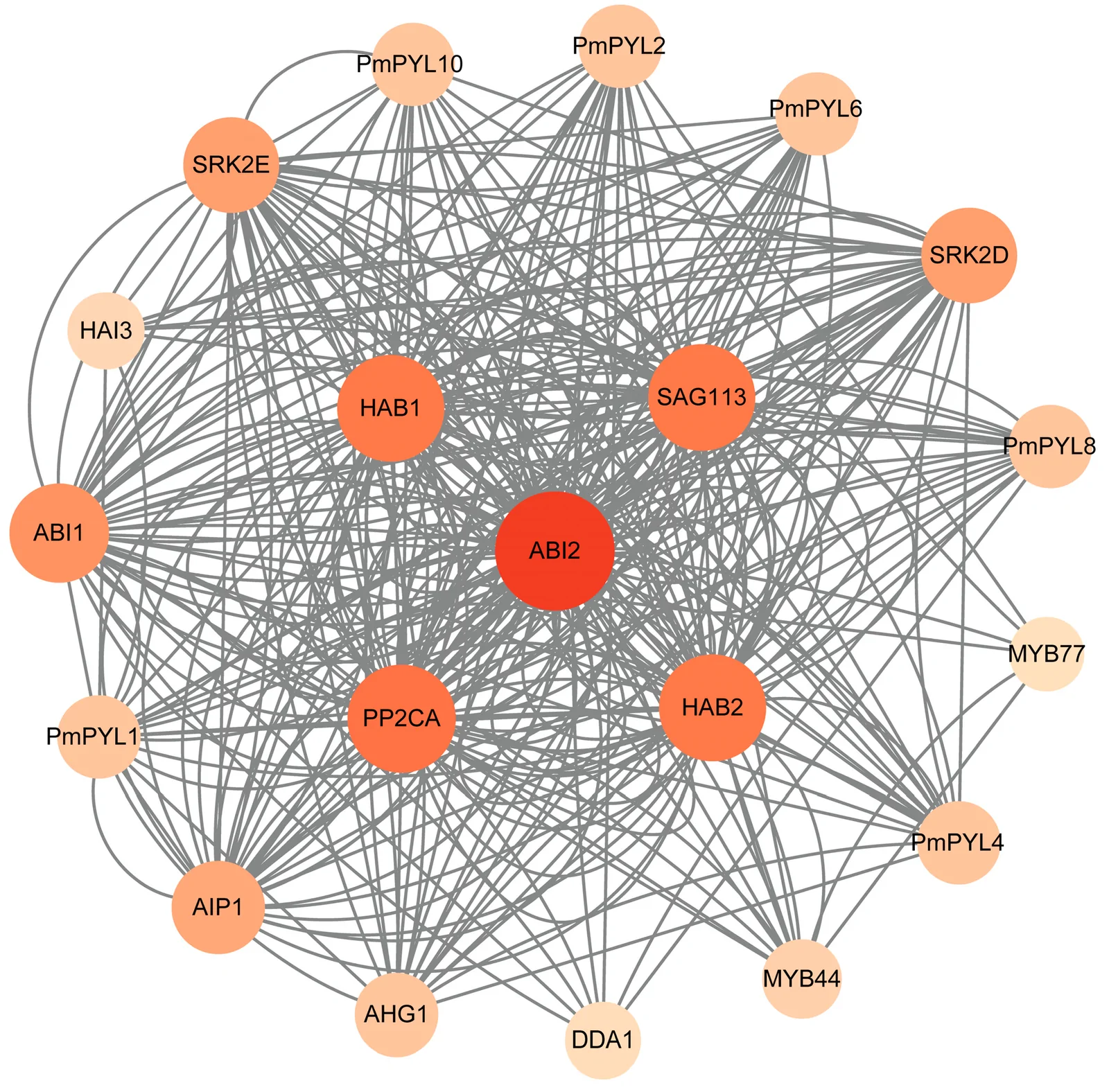
Predicted PmPYL-associated protein interaction network in broomcorn millet. The network was inferred from orthologous interactions of *Arabidopsis thaliana* PYL receptors and ABA signaling-related components using STRING. Edges represent predicted protein associations rather than experimentally validated interactions. Node size and color intensity indicate node degree, with larger and darker nodes representing proteins with more connections.

The *Arabidopsis* reference network connected PYL receptors with several ABA-signaling-associated proteins, including clade A PP2Cs and SnRK2-related kinases (Figure 7). Sequence comparisons identified candidate *Arabidopsis* PYL matches for six PmPYL proteins (Table S8). Because the network associations originate from *Arabidopsis* and the sequence matches do not resolve all one-to-one orthologous relationships, these results identify possible interaction candidates for future testing rather than confirmed PmPYL protein partners.

### 3.9. Tissue-Specific Expression Patterns of PmPYL Genes

**Figure 8:**
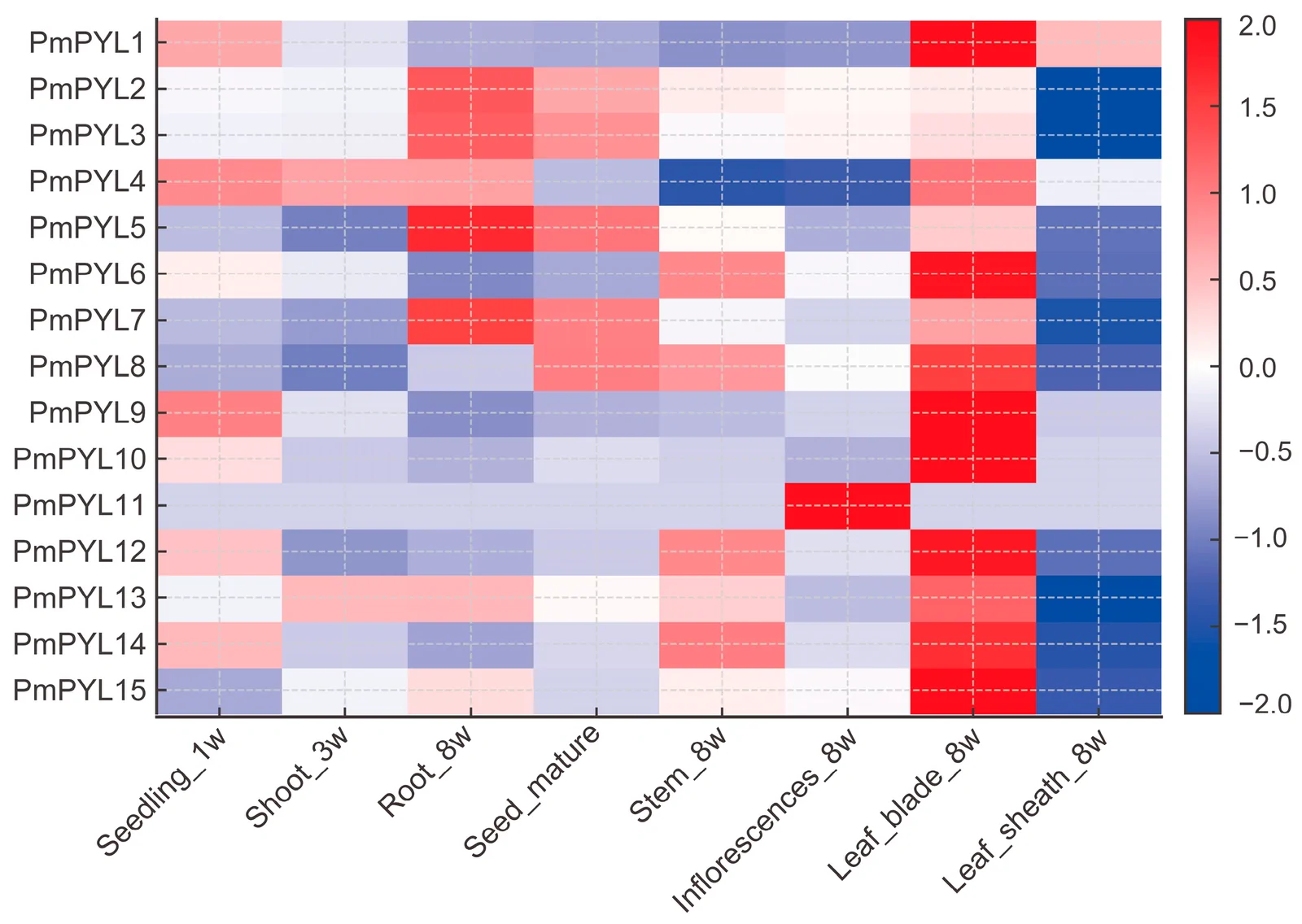
Tissue-expression profiles of PmPYL gene. RPKM values were transformed as log_2_ (RPKM + 1) and standardized separately for each gene across the eight tissues or developmental stages. The color scale shows the resulting within-gene z-scores: red indicates expression above the gene’s eight-context mean, white indicates expression near the mean, and blue indicates expression below the mean.

To characterize the spatial expression patterns of the *PmPYL* gene family, transcript abundance of the 15 *PmPYL* genes was examined across eight broomcorn millet tissues or developmental stages (Figure 8). Considerable variation in expression patterns was observed among family members.

Several genes, including *PmPYL2*, *PmPYL3*, *PmPYL5*, and *PmPYL7*, showed relatively broad expression across multiple vegetative tissues, particularly leaf blade, leaf sheath, stem, and root. *PmPYL2* exhibited pronounced expression in leaf blade, leaf sheath, and root, whereas *PmPYL3* and *PmPYL7* showed similarly broad expression across several vegetative tissues.

In contrast, several *PmPYL* genes displayed more restricted expression patterns. *PmPYL11* showed preferential expression in inflorescences, whereas *PmPYL10* exhibited relatively high expression in roots compared with the other examined tissues. *PmPYL1*, *PmPYL4*, *PmPYL8*, and *PmPYL9* generally showed low transcript abundance across most tissues, although relatively higher expression was observed in individual tissues.

Across the seven confirmed A/B pairs, mean promoter Jaccard dissimilarity was 0.527 (range, 0.313–0.647), whereas the mean tissue-expression correlation was 0.860 (range, 0.663–0.994; Table S5). The observed pairs showed lower mean promoter dissimilarity and higher mean expression correlation than alternative within-group A/B mappings (24 exact mappings; nominal one-sided *p* = 0.0417 for each metric), but neither comparison remained significant after Holm correction (adjusted *p* = 0.0833). These exploratory results indicate pair-dependent regulatory variation alongside generally conserved tissue-expression profiles; they do not establish functional subfunctionalization.

### 3.10. Stress-Responsive Expression Profiles of PmPYL Genes

**Figure 9:**
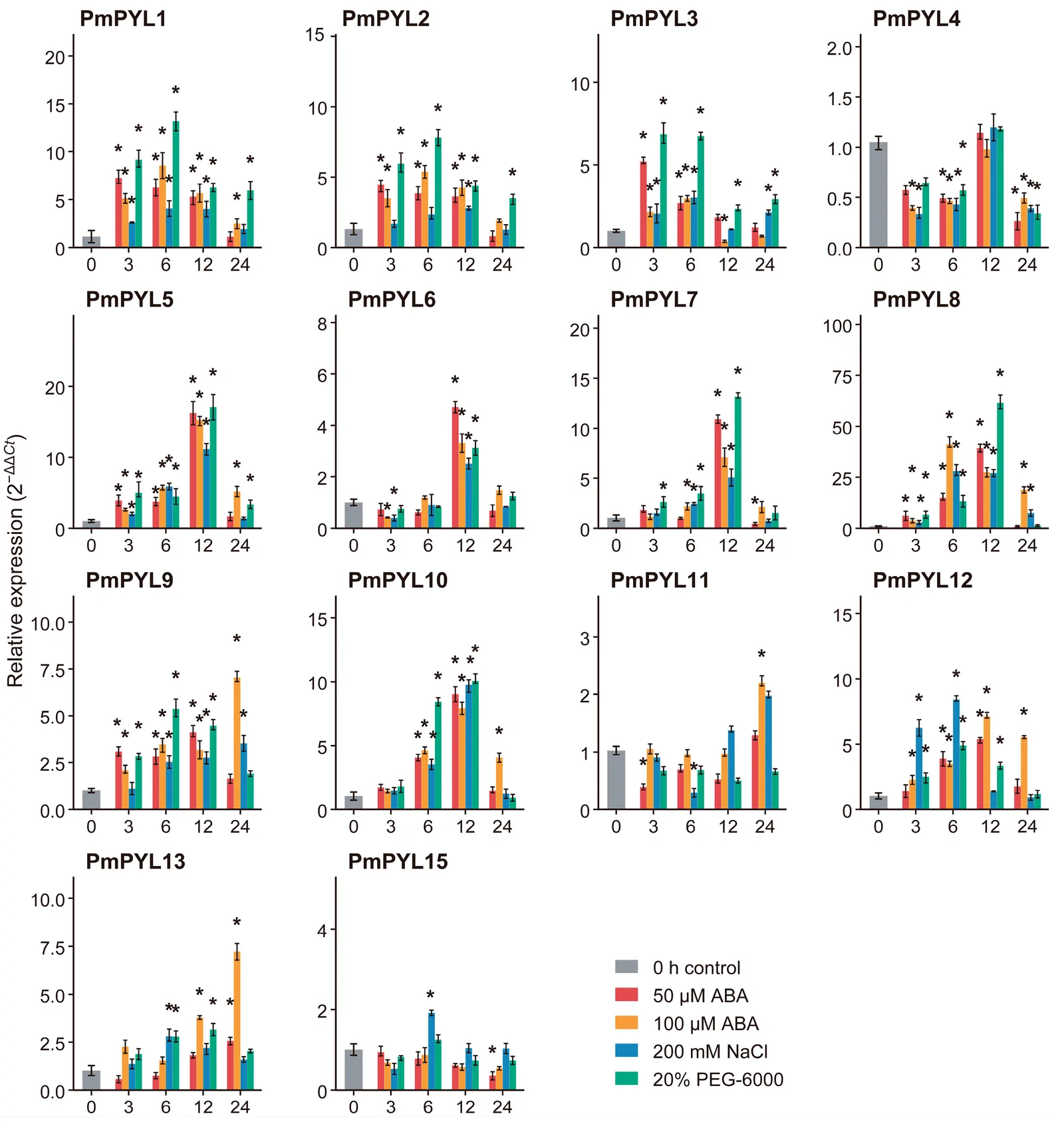
Expression profiles of *PmPYL* genes in broomcorn millet following ABA, NaCl, and PEG-6000 treatments. Expression was normalized to *PmActin* and calculated by the 2^−ΔΔCt^ method using the mean ΔCt of the three shared pretreatment (0 h) samples as the gene-specific calibrator. Bars show mean ± SD of three biological replicates. An asterisk indicates an adjusted *p* < 0.05 for a two-sided Dunnett comparison with that gene’s shared 0 h group; adjustment covers the 16 treatment–time comparisons within each gene. Statistical tests were performed on ΔCt values.

The expression profiles of 14 *PmPYL* genes in seedling leaves varied across treatments and sampling times (Figure 9). In the two-way ANOVA of post-treatment ΔCt values, the treatment effect was significant for 13 genes, whereas the time effect and treatment × time interaction were significant for all 14 genes (*p* < 0.05). The treatment main effect was not significant for *PmPYL4* (*p* = 0.474). In comparisons with the shared pretreatment baseline, 133 of the 224 treatment–time combinations were significant after Dunnett adjustment within genes.

*PmPYL8* showed the largest change in relative expression, reaching 61.45 ± 10.50 under PEG-6000 at 12 h. It also showed marked responses to ABA and NaCl. *PmPYL10* responded to several treatments, reaching 10.06 ± 1.25 under PEG-6000 at 12 h, while *PmPYL12* reached 8.41 ± 2.85 under NaCl at 6 h. These examples illustrate differences in both the magnitude and timing of the observed expression changes.

Other genes exhibited more restricted or variable profiles. *PmPYL3* increased early under PEG-6000, whereas *PmPYL4* showed no significant treatment main effect despite differences from 0 h at individual treatment–time combinations. *PmPYL14* was excluded because reliable qRT-PCR data were unavailable. Because no time-matched untreated controls were included, these comparisons establish differences from the pretreatment baseline but cannot isolate treatment effects from changes associated with elapsed time.

## 4. Discussion

### 4.1. Evolutionary Conservation and Regulatory Diversification of the PmPYL Family

Broomcorn millet is an allotetraploid species whose genome retains extensive homoeologous relationships between its two constituent subgenomes. Previous genomic studies have demonstrated substantial retention of duplicated genes following polyploidization, accompanied by asymmetric gene loss during subsequent diploidization [6,39]. In the present study, 15 PmPYL genes were identified, with eight located in the A subgenome and seven in the B subgenome. Seven synteny-supported A/B homoeologous pairs were identified, accounting for 14 of the 15 family members, indicating substantial retention of homoeologous PYL genes following allopolyploidization. In contrast, the five additional non-homoeologous collinear relationships could not be confidently attributed to independent segmental-duplication events based on collinearity alone. No tandem duplication involving two identified PmPYL members was detected. These findings indicate that homoeolog retention is an important feature of the present-day PmPYL family, rather than establishing independent segmental duplication as its predominant expansion mechanism.

The strong conservation of PmPYL protein architecture further supports evolutionary constraints on this receptor family. The revised maximum-likelihood phylogeny revealed conserved relationships between PmPYL proteins and their homologs in other grass species. The reported Ka/Ks ratios of all 11 collinear PmPYL relationships were below 1.0, consistent with predominantly purifying selection, although estimates for highly divergent gene pairs require cautious interpretation. This evolutionary conservation is biologically plausible because PYL receptors rely on conserved structural features for ABA perception and PP2C recognition. Structural studies have demonstrated that ABA-induced rearrangement of the conserved gate and latch loops creates the receptor surface required for PP2C engagement, providing a mechanistic explanation for the conservation of the receptor core [42].

At the same time, conservation of the receptor core does not imply complete functional redundancy. Comparative analysis of PYL receptors from 55 plant genomes has revealed substantial functional diversification despite strong conservation of their overall structural framework [43]. More generally, duplicated genes can be preserved through partitioning of ancestral regulatory functions even when their protein-coding sequences remain highly constrained, a process described by the duplication–degeneration–complementation model [44]. Descriptive comparisons of the seven A/B homoeologous pairs revealed variation in promoter cis-element composition and expression magnitude despite generally conserved expression profiles. However, these observations remain exploratory because the comparisons were not statistically significant after multiple-testing correction and the public tissue-expression matrix lacked biological-level replication. Therefore, the available evidence does not establish regulatory subfunctionalization.

The molecular-dating results should nevertheless be interpreted cautiously. Using the grass reference synonymous substitution rate of 6.5 × 10^−9^ substitutions per synonymous site per year, following Sun et al. [39], the 11 gene pairs retained for molecular dating yielded approximate divergence-time estimates ranging from 5.1 to 104.9 MYA. However, these estimates depend on the assumed substitution rate and should not be regarded as precise dates of individual duplication events. In particular, PmPYL11–PmPYL13 exhibited a high Ks value (3.3386), at which synonymous-site saturation may compromise reliable molecular dating; therefore, no absolute divergence-time estimate was assigned to this pair. Moreover, pairwise gene-sequence divergence should not be equated with the timing of allotetraploidization, because divergence between ancestral subgenomes and their subsequent merger represent distinct evolutionary events.

### 4.2. Variation in Predicted Promoter Features and Expression Patterns of PmPYL Genes

Although PmPYL proteins are structurally conserved, their predicted regulatory regions and expression profiles differ. The promoters contain varying combinations of hormone-, stress-, light-, defense-, and development-associated cis-elements. Their predicted numbers alone did not explain the qRT-PCR profiles: *PmPYL4* contained the largest number of predicted stress-associated elements, yet its treatment main effect was not significant in the post-treatment analysis. Motif predictions therefore provide candidates for regulatory investigation but cannot establish which elements are active under the conditions tested.

Tissue-expression patterns provide another indication of variation within the family. *PmPYL2*, *PmPYL3*, *PmPYL5*, and *PmPYL7* were expressed across several vegetative tissues, whereas *PmPYL11* showed preferential expression in inflorescences and *PmPYL10* had relatively high expression in roots. Receptor-specific expression and function have also been described in *Arabidopsis* [20,45]. The present tissue data support differences in where *PmPYL* genes are expressed, although they do not establish corresponding physiological functions in broomcorn millet.

Similar variation has been reported for PYL families in other species. In maize, individual *ZmPYL* genes showed different responses to ABA and dehydration, including contrasting expression patterns between leaves and roots [46]. In a separate study, heterologous overexpression of *ZmPYL8*, *ZmPYL9*, or *ZmPYL12* increased drought resistance in *Arabidopsis* [47]. In rice, constitutive overexpression of *OsPYL/RCAR5* enhanced drought and salt tolerance but inhibited growth [21]. These findings show that variation among PYL family members can extend beyond expression patterns to experimentally measurable physiological effects. They provide a context for investigating *PmPYL* genes, without establishing equivalent functions in broomcorn millet.

The revised qRT-PCR analysis shows that the genes also differ in their expression trajectories after the experimental treatments. *PmPYL8* had the greatest observed expression increase, particularly under PEG-6000 at 12 h, and showed substantial changes under ABA and NaCl. *PmPYL10* and *PmPYL12* responded at several treatment–time combinations, while *PmPYL3* showed an early increase under PEG-6000. These differences identify *PmPYL8, PmPYL10*, and *PmPYL12* as candidates for further study, but transcript abundance does not establish receptor activity or a positive effect on stress tolerance [21,22,48].

The experimental design also limits the interpretation of treatment specificity. A shared 0 h sample provides a pretreatment baseline, but the absence of time-matched untreated or mock controls means that treatment-associated changes cannot be separated conclusively from circadian, developmental, irrigation-related, or other time-dependent variation. The ABA treatment additionally lacked a matched 0.1% ethanol vehicle control, and cultivation trays were not randomly allocated. Accordingly, the expression profiles are best treated as exploratory evidence. Experiments with time-matched controls, appropriate vehicle controls, randomized allocation, and functional assays are needed to test whether these genes contribute to ABA responses and stress adaptation.

### 4.3. A Conserved ABA Signaling Core Embedded in Multiple Regulatory Layers

The predicted protein association network is consistent with conservation of the canonical PYL–PP2C–SnRK2 signaling module in broomcorn millet. Several PmPYLs were associated with clade A PP2Cs, including ABI1, ABI2, HAB1, PP2CA/HAB2, AHG1, and HAI3. This agrees with the established ABA signaling mechanism in which ABA-bound PYL receptors inhibit PP2Cs and thereby release SnRK2 kinases from phosphatase-mediated repression [13,14,42]. However, because the present network was inferred by interolog prediction, these associations should be regarded as computationally supported rather than experimentally confirmed interactions.

PYL receptor activity is also regulated beyond transcription. TOR-dependent phosphorylation can suppress ABA receptor activity and contribute to growth–stress balancing, whereas CARK1 and the CBL1/9–CIPK1 module regulate receptor activity through phosphorylation in opposite directions [47,48]. In addition, ubiquitination-mediated turnover of receptors such as PYR1 and PYL4 provides another mechanism controlling receptor abundance [49]. These findings indicate that ABA perception is fine-tuned through multiple post-translational mechanisms rather than being determined solely by receptor transcript levels.

Overall, the PmPYL family appears to combine a highly conserved ABA-perception core with substantial regulatory diversification. Protein sequence conservation preserves the basic receptor framework, whereas promoter divergence, tissue-specific expression, stress-responsive transcription, and multiple post-translational regulatory mechanisms may provide additional flexibility for receptor-specific signaling. This multilayered regulatory architecture may help explain how a structurally conserved receptor family can participate in diverse developmental and stress responses in broomcorn millet.

## 5. Conclusions

This study identified 15 *PmPYL* genes in broomcorn millet, comprising eight A-subgenome and seven B-subgenome members. Sequence, structural, and syntenic analyses revealed a conserved receptor framework with seven retained A/B homoeologous pairs, while low Ka/Ks ratios were consistent with purifying selection acting on protein-coding sequences. Family members differed in predicted promoter composition and tissue-expression patterns. In seedling leaves, qRT-PCR analysis revealed changes in transcript abundance relative to the shared pretreatment baseline following ABA, NaCl, and PEG-6000 treatments, with *PmPYL8* showing the largest observed increase. These expression profiles identify *PmPYL8*, *PmPYL10*, and *PmPYL12* as candidates for further investigation. Their roles in ABA signaling and stress adaptation require experimental validation.

## Figures and Tables

**Table 1:** Genomic and physicochemical characteristics of the *PmPYL* gene family in broomcorn millet.

Gene ID	Gene Name	Chr	Start	End	Amino Acid Number	Theoretical pI	Molecular Weight (Da)
*PmPYL1*	*AJ81AG08520.1*	1A	5,778,067	5,779,822	197	6.39	21,469.10
*PmPYL2*	*AJ81AG10060.1*	1A	7,210,812	7,214,614	213	6.60	24,023.26
*PmPYL3*	*AJ81BG08520.1*	1B	13,165,125	13,168,905	214	6.42	24,020.2
*PmPYL4*	*AJ81BG11880.1*	1B	17,219,082	17,220,854	198	6.39	21,559.18
*PmPYL5*	*AJ83AG05420.1*	3A	3,416,738	3,419,823	206	6.51	22,311.18
*PmPYL6*	*AJ83AG20440.1*	3A	12,710,323	12,711,548	202	8.16	21,533.27
*PmPYL7*	*AJ83BG06600.1*	3B	4,275,144	4,276,748	206	6.34	22,182.06
*PmPYL8*	*AJ83BG22830.1*	3B	15,182,639	15,183,247	202	8.40	21,520.28
*PmPYL9*	*AJ84AG09710.1*	4A	7,127,674	7,128,523	204	7.60	21,956.61
*PmPYL10*	*AJ84BG11120.1*	4B	9,977,748	9,978,666	199	6.79	21,505.12
*PmPYL11*	*AJ85AG09990.1*	5A	6,004,052	6,004,642	196	6.07	21,260.97
*PmPYL12*	*AJ89AG28230.1*	9A	39,357,501	39,359,537	210	5.08	22,531.31
*PmPYL13*	*AJ89AG39760.1*	9A	47,656,891	47,658,178	223	6.89	23,211.21
*PmPYL14*	*AJ89BG31240.1*	9B	48,823,144	48,824,969	209	5.08	22,388.08
*PmPYL15*	*AJ89BG43770.1*	9B	59,403,776	59,404,447	209	6.41	21,594.31

**Table 2:** Predicted secondary-structure composition and subcellular localization of PmPYL proteins.

Gene ID	Helix %	Extended Strand %	Coiled %	Subcellular Localization
*PmPYL1*	29.44	31.47	39.09	Cytoplasm
*PmPYL2*	33.33	25.82	40.85	Cytoplasm
*PmPYL3*	33.64	25.23	41.12	Cytoplasm
*PmPYL4*	27.27	33.33	39.39	Nucleus/Cytoplasm
*PmPYL5*	31.55	25.24	43.20	Cytoplasm
*PmPYL6*	32.67	32.67	34.65	Chloroplast
*PmPYL7*	33.50	26.21	40.29	Cytoplasm
*PmPYL8*	32.18	33.17	34.65	Chloroplast
*PmPYL9*	32.84	34.31	32.84	Chloroplast/Cytoplasm
*PmPYL10*	32.66	36.18	31.16	Chloroplast/Cytoplasm
*PmPYL11*	33.67	33.67	32.65	Cytoplasm
*PmPYL12*	34.29	28.10	37.62	Cytoplasm
*PmPYL13*	31.84	34.98	33.18	Extracellular/Vacuole
*PmPYL14*	30.14	27.27	42.58	Chloroplast
*PmPYL15*	29.67	33.49	36.84	Extracellular/Vacuole

**Table 3:** Ka/Ks ratios and estimated divergence times of duplicated *PmPYL* gene pairs in broomcorn millet.

Gene Pair	Classification	Ka	Ks	Ka/Ks	MYA	Type of Selection
*PmPYL1–PmPYL4*	A/B homoeologous	0.0241	0.2879	0.0835	22.1	Purifying
*PmPYL2–PmPYL3*	A/B homoeologous	0.0057	0.0659	0.0867	5.1	Purifying
*PmPYL5–PmPYL7*	A/B homoeologous	0.0077	0.0914	0.0839	7.0	Purifying
*PmPYL6–PmPYL8*	A/B homoeologous	0.0092	0.2045	0.0449	15.7	Purifying
*PmPYL9–PmPYL10*	A/B homoeologous	0.0071	0.2376	0.0297	18.3	Purifying
*PmPYL12–PmPYL14*	A/B homoeologous	0.0338	0.2164	0.1563	16.6	Purifying
*PmPYL13–PmPYL15*	A/B homoeologous	0.0051	0.3081	0.0166	23.7	Purifying
*PmPYL1–PmPYL9*	Non-homoeologous	0.1343	0.8954	0.1500	68.9	Purifying
*PmPYL1–PmPYL10*	Non-homoeologous	0.1429	1.1210	0.1274	86.2	Purifying
*PmPYL4–PmPYL9*	Non-homoeologous	0.1221	1.1249	0.1085	86.5	Purifying
*PmPYL4–PmPYL10*	Non-homoeologous	0.1259	1.3643	0.0923	104.9	Purifying

## Data Availability

The data presented in this study are available within the article and its Supplementary Materials. The analysis scripts are available from the corresponding author upon reasonable request.

## Supplementary Materials

The following supporting information can be downloaded at: https://www.mdpi.com/article/10.3390/biology15211944/s1, Table S1. BLASTP matches used to identify AJ8 PmPYL candidates. Table S2. HMMER confirmation of the PF10604 domain in 15 PmPYL proteins. Table S3. Protein sequences of the 15 identified PmPYL genes. Table S4. MAFFT alignment of the 59 PYL protein sequences used for phylogenetic analysis. Table S5. Synteny-supported AB homoeologous PmPYL pairs and regulatory comparisons. Table S6. Predicted cis-regulatory elements in PmPYL promoters by functional category. Table S7. Published tissue-expression values and gene-ID mapping for PmPYL genes. Table S8. Sequence-based Arabidopsis PYL matches for the six PmPYL proteins. Table S9. Primers used for qRT-PCR analysis of PmPYL genes and PmActin. Table S10. Biological-replicate Ct values and calculated relative expression of PmPYL genes. Figure S1. Phylogenetic relationships among the 15 PmPYL proteins in broomcorn millet.
