# Supplementary material for: Genome-Wide Identification and Characterization of the ABA Receptor PYL Gene Family in Broomcorn Millet (Panicum miliaceum L.)
Source: Biology (Basel). 2026 Oct 27;15(21):1944. doi: 10.3390/biology15211944 (PMC13761657; doi:10.3390/biology15211944)
Supplement: Supplementary file 1 [file biology-15-01944-s001.zip › Figure S1.pdf]

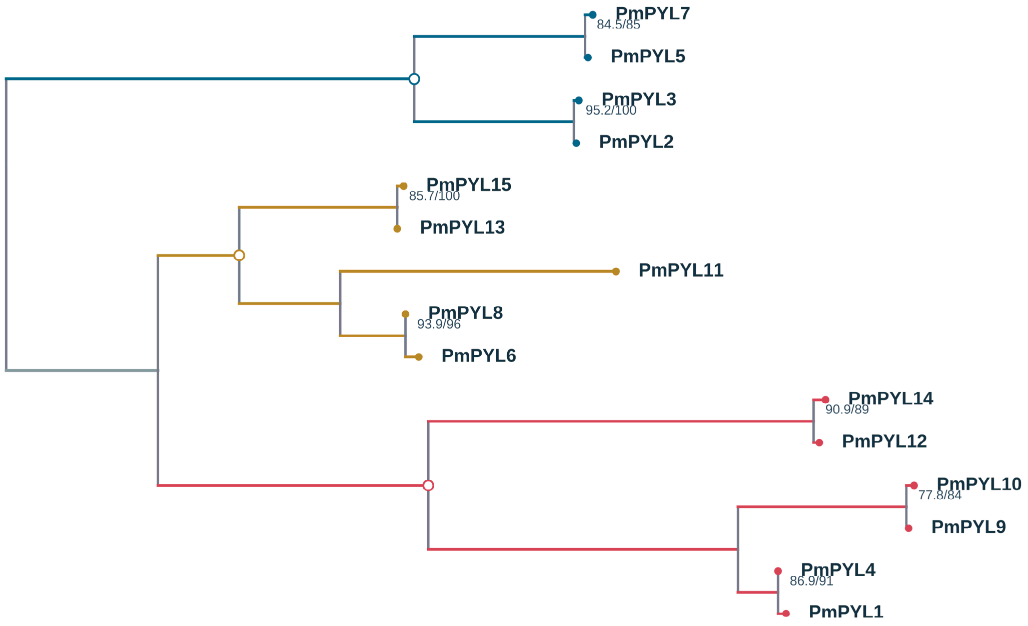

Figure S1. Phylogenetic relationships among the 15 PmPYL proteins in broomcorn millet. The PmPYL relationships were extracted from the maximum-likelihood phylogeny of 59 PYL proteins presented in Figure 2A. Numbers at selected nodes indicate SH-aLRT/ultrafast bootstrap support values obtained from the original 59-protein ML analysis, each based on 1000 replicates.
